# Arrhythmias in Dengue: Moving Upstream from Electrocardiographic Findings to Cardio-Hemodynamic Dysfunction. Comment on López-Delgado et al. Arrhythmias in Dengue: A Systematic Review and Meta-Analysis. *Pathogens* 2026, *15*, 497

**DOI:** 10.3390/pathogens15070699

**Published:** 2026-07-02

**Authors:** Muhammad Iqhrammullah, Derren D. C. H. Rampengan

**Affiliations:** 1 Postgraduate Program of Public Health, Universitas Muhammadiyah Aceh, Banda Aceh 23247, Indonesia; 2 Faculty of Medicine, Universitas Sam Ratulangi, Manado 95115, Indonesia; derrenrmed@gmail.com

We read with great interest the systematic review and meta-analysis by López-Delgado et al., which synthesized available evidence on arrhythmias in dengue [1]. The authors included 35 studies involving 6948 patients and reported that all cardiac arrhythmia occurred in approximately one-quarter of patients with dengue. The most frequent abnormalities were sinus bradycardia and sinus tachycardia, whereas atrioventricular block was uncommon. 

However, we believe that the findings of this meta-analysis should be interpreted through a broader upstream cardio-hemodynamic framework. The pooled estimate of arrhythmias was largely driven by sinus rate abnormalities. These findings are clinically relevant, but they may not always represent primary electrical disease. Sinus tachycardia may reflect fever, pain, dehydration, plasma leakage, bleeding, anemia, or evolving shock. Sinus bradycardia may reflect autonomic changes, defervescence, or relative bradycardia during acute viral illness. Thus, many electrocardiographic abnormalities in dengue may be downstream markers of upstream physiological disturbance rather than isolated arrhythmic events.

This distinction is important because blood pressure, pulse pressure, and overt shock are also downstream clinical expressions. Earlier cardiovascular changes may occur at the level of intravascular volume, venous return, ventricular filling, myocardial performance, stroke volume, systemic vascular resistance, and autonomic regulation. Therefore, the key clinical question is not only “how often do arrhythmias occur in dengue?”, but also “what upstream cardiovascular processes produce these rhythm abnormalities, and which of them are clinically meaningful?”.

In Vietnamese patients with different dengue severity grades, severe dengue was associated with systolic and diastolic myocardial impairment [1]. Severe dengue patients had increased left myocardial performance index, reduced septal systolic velocity, reduced right ventricular systolic velocity, and reduced early diastolic velocity. These abnormalities improved by discharge, suggesting transient dengue-related myocardial dysfunction [1]. Similarly, a study involving pediatric cohort with dengue shock syndrome grade 4 found that decreased preload from bleeding and vascular leakage played a central role in shock [2]. However, decreased stroke volume and low systemic vascular resistance were also identified as additional contributors [3]. This finding suggests that dengue shock is not only a blood pressure problem. It is a broader hemodynamic disorder involving preload, stroke volume, vascular tone, and compensatory cardiac responses.

Population-level evidence also strengthens the clinical relevance of dengue-associated cardiovascular involvement. A study recently reported a national population-based cohort study in Singapore, including 65,207 adults with dengue virus infection and 1,616,865 population-based controls [4]. Acute dengue was associated with higher odds of any cardiovascular event, major adverse cardiac events, ischemic heart disease, and dysrhythmia within 30 days [4]. Dysrhythmia showed a particularly strong association. However, the absolute excess burden was modest, generally fewer than one excess cardiovascular event per 100 dengue cases, except among adults aged 60 years or older [4].

Collectively, these findings suggest that dengue-associated arrhythmias should not be viewed only as isolated electrocardiographic endpoints. A more useful interpretation is that rhythm abnormalities may represent downstream signals of upstream cardio-hemodynamic stress. This upstream framing has several practical implications. First, ECG abnormalities in dengue should be interpreted according to disease phase and clinical context. A sinus tachycardia during the critical phase may have a different meaning from sinus tachycardia during fever alone. Similarly, sinus bradycardia during defervescence may be benign, whereas bradycardia with hypotension, syncope, electrolyte disturbance, or suspected myocarditis may require closer evaluation.

Second, cardiac assessment should not rely only on blood pressure or a single ECG. A patient may maintain blood pressure despite reduced preload, impaired ventricular relaxation, or early myocardial dysfunction. In higher-risk patients, particularly those with warning signs, severe dengue, abnormal ECG findings, chest pain, syncope, unexplained hypotension, electrolyte abnormalities, suspected myocarditis, or older age, ECG should be integrated with hemodynamic and myocardial assessment. This may include echocardiography, inferior vena cava collapsibility assessment, troponin, electrolyte evaluation, hematocrit trends, platelet trends, pleural effusion assessment, and WHO severity classification.

Third, future studies should separate sinus rate abnormalities from clinically significant arrhythmias. Sinus bradycardia, sinus tachycardia, atrioventricular block, atrial fibrillation or flutter, supraventricular tachyarrhythmias, ventricular arrhythmias, QT abnormalities, and ST-T changes should be reported separately. This distinction would help avoid overestimating the burden of clinically dangerous rhythm disease while preserving the value of ECG abnormalities as warning signals.

Fourth, future research should link rhythm abnormalities with clinically meaningful outcomes. These include shock, fluid overload, need for inotropes, myocarditis, intensive care admission, prolonged hospitalization, mortality, and post-acute cardiovascular sequelae. Without such outcome linkage, the clinical importance of pooled arrhythmia prevalence remains uncertain.

The meta-analysis by López-Delgado et al. is therefore an important starting point. It answers the “how common” question. The next step is to address the “why it happens” and “when it matters” questions. We suggest that dengue-associated arrhythmias should be interpreted within an upstream model: dengue virus infection leads to immune activation and vascular permeability, followed by plasma leakage, altered preload, transient myocardial dysfunction, autonomic imbalance, and systemic inflammatory stress. ECG abnormalities then emerge as downstream markers of this process. Regardless, integrating ECG findings with echocardiography, intravascular volume assessment, biomarkers, severity staging, and patient-centered outcomes may improve risk stratification and help distinguish benign transient rhythm changes from clinically meaningful cardiac involvement.

## Abbreviation

The following abbreviation is used in this manuscript:

ECG	Electrocardiogram
